# Lung injury following butane inhalation

**DOI:** 10.36416/1806-3756/e20250364

**Published:** 2026-03-05

**Authors:** Luciana Volpon Soares Souza, Arthur Soares Souza, Edson Marchiori

**Affiliations:** 1. Ultra X, São José do Rio Preto (SP) Brasil.; 2. Faculdade de Medicina de Rio Preto, São José do Rio Preto (SP) Brasil.; 3. Universidade Federal do Rio de Janeiro, Rio de Janeiro (RJ) Brasil.

**Figure 1:**
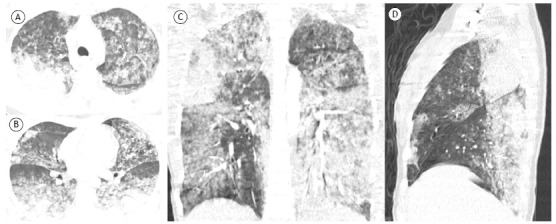
Axial chest CT images (in A and B), as well as coronal and sagittal chest CT images (in C and D, respectively), showing areas of consolidation and ground-glass attenuation distributed diffusely throughout the lungs.

A 17-year-old female inhaled butane from a portable air horn during a party and developed acute respiratory failure immediately after inhalation. She was near a hospital and was treated immediately. She developed respiratory arrest upon arrival and received advanced life support. She was intubated, and pink frothy fluid was suctioned from the endotracheal tube. Her clinical status improved, and she underwent chest CT examination, which showed extensive areas of consolidation and ground-glass attenuation distributed diffusely throughout the lungs (Figure 1). She stayed in the ICU for 9 days and was discharged after recovery. 

Butane is an odorless, colorless gas commonly used as a solvent and as a household fuel. Its liquefied form is used in cigarette lighters, camping stoves, gas lamps, spray paint, and some air fresheners and deodorants, as well as in gasoline and other fuels.^1^ ^-^ ^3^ In Brazil, portable air horns are widely used at parties during events such as the World Cup and Carnival. They are sold freely and are increasingly used by young people as inhalants. Common lung findings in autopsy cases include pulmonary congestion, pulmonary edema, and alveolar hemorrhage. Death is usually caused by cardiac involvement, respiratory center depression, or brain injury.^1^ ^,^ ^2^
